# Foreign

**Published:** 1840-10-24

**Authors:** 


### FOREIGN.

*Practical observations on peculiar affections of the throat, arising from abscess between the pharynx and spine, and occurring in children and adults; exemplified by cases.* By Christopher Fleming, M. D.

[Concluded from p. 675.]

Let us now inquire into those circumstances which will best explain the nature of the affection, its progress, and the treatment calculated to remedy it.

From the report of the cases, and the details of the attendant symptoms (given perhaps with unnecessary minuteness,) a particular description of this affection may in a great measure be dispensed with. It is evidently one of an inflammatory character, and, like the majority of such, may be acute or chronic, circumscribed or diffused, as appears from the following extract from the "Elements of the Practice of Medicine," by Drs. Bright and Addison: "Acute idiopathic pharyngitis, or that in which the inflammation is limited to the pharynx, is of extremely rare occurrence. We have only seen two instances of the kind. One occurred in a female beyond the middle period of life, the other in a man between forty and fifty years of age. The female, after exposure to cold, was attacked with pain in the throat, and great pain and difficulty in swallowing, speedily followed by the ordinary signs of febrile excitement. The pain and difficulty in swallowing rapidly increased, till at length the smallest particle of food or drink could not be taken. *The voice was distinct, but the articulation imperfect, as if the patient were unable or unwilling to exert the laryngeal muscles.* On making a careful inspection, scarcely the slightest trace of inflammation could be detected in the throat, nor could the epiglottis be distinguished; but on making pressure on one spot externally on the right side, and at the posterior part of the thyroid cartilage, the patient complained of acute pain. She was bled from the arm, and had leeches applied to the neck, followed by a large warm poultice, and the inhalation of warm water vapour. Under such treatment the disease yielded in a few days to such an extent that she was again able to swallow; but acute pleurisy now supervened, which, in her reduced state and bad constitution, speedily proved fatal. On examining the parts after death, unequivocal marks of acute inflammation were found at the lower part of the pharynx, the inferior portion of the epiglottis, and posterior surface of the arytenoid cartilages, together with such a degree of purulent infiltration into the submucous cellular tissue in the latter situation, as almost to present the appearance of an abscess, although the cellular structure itself was not broken down. Had not the disease been subdued, this might have undoubtedly passed into a state of abscess, which, by its increase of size and consequent pressure upon the larynx, would probably have seriously interfered with the process of respiration, or even have proved fatal by producing suffocation. The symptoms of the other case bore an exact resemblance to the above, with the exception of the pain on making pressure externally, which was not present in that instance."

Here are evidently recognizable the local phenomena of diffuse inflammation confirmed by the transfer of disease to the thoracic organs, so frequent in similar cases.

For my own part, I am disposed to the opinion, that, with very few exceptions, this particular affection of the throat is always symptomatic, in some cases, of direct injury, as in that cited from Sir Astley Cooper's Lectures; but, in by far the greater majority of cases, symptomatic of some constitutional derangement, general or specific; general, as the result of fever, and particularly of that form of fever termed by the French pathologists "gastro-encéphalite," or specific, in the two-fold sense in which that term is applied by practical authors, namely, specific as to the exciting cause, or specific as to modification by constitutional peculiarity.

These considerations appear to me of great importance in influencing the character and progress of the attack; in the one, stamping it with an acuteness and rapidity of supervention alarmingly deceptive, and in the other, with a chronic tediousness not likely to escape detection.

Its seat is unquestionably between the back of the pharynx and the muscles on the anterior part of the spine, in the loose cellular, or rather reticular texture, there to be found intervening. It is nothing more or less than inflammation in this region, terminating in suppuration; and, to have an accurate knowledge of the pathological conditions present, as well as of the symptoms attendant on them, and the requisite treatment, it is absolutely necessary to bear in recollection both the structural and relative anatomy of the pharynx. Perhaps in few other lesions can we more satisfactorily exemplify the relations between cause and effect. For example, the extreme tension, and almost unyielding hardness of those tumors on pressure, stated in the report of the cases, will be accounted for by the very strong, though apparently diaphanous membrane, upon which its superior constrictor is expanded at its posterior and upper part; and the locked state of the jaws will be partially accounted for, at all events, by the attachment of a portion of that muscle in its lateral aspect. The facility of opening the mouth in some cases, and the impossibility in others, may be referrible either to the amount of matter collected, and hence greater tension, or perhaps to the formation of the lower jaw in the child, as it was in the youngest that this freedom of separation existed most. Again, the cerebral symptoms, and those affecting the respiratory organs, are easily attributable to the mechanical pressure on the nerves and important vessels, on the one hand, and to the presence of impure blood in the nervous centre, on the other; while the peculiar position of the head, the inability to elevate it, the rigid state of the muscles at the back of the neck, and the supervention of the almost apoplectic interval when in the recumbent posture, may be referred to the varying pressure exercised on the glottis from the projection of the abscess opposite that opening, necessarily influencing more or less the free entrance of air to the lungs.

In childhood these effects on the brain are of the greatest moment; all practitioners are aware of the great predisposition which exists at that period of life to cerebral disease; that it is induced by most trifling, sometimes the most opposite, causes, and that none more frequently give rise to it than those which create any derangement in the circulating capillary system, already so necessarily active in the immediate vicinity of this organ; that the several cutaneous diseases incidental to this period of life often terminate fatally in it, or are complicated with it, and none perhaps more than those which are accompanied with affections of the throat; for instance, variola or scarlatina. How awfully fatal are not those diseases in childhood; and in the most alarming cases, is not the throat seriously engaged? Nay more, may it not be one of the causes of cerebral affection? These considerations have led me to an opinion which I am strongly induced to entertain, from reflections both anatomical and practical; namely, that the more close investigation of the causes and symptoms of this affection in its *acute* form may tend to limit the fatal results of those diseases, inasmuch as in them we know it to be a fact, that the glands in the neighborhood of the throat are constantly and extensively implicated in its inflammatory affections; and that if, as I at present feel satisfied from the examinations I have made, some of those glands are to be found in the loose reticular texture between the pharynx and the spine, more frequently in the earlier than the advanced periods of life, we have an additional point, at all events, to direct our attention and treatment to, which may assist materially the operation of remedies. That this affection which I bring under the consideration of the profession is not unfrequently an acute inflammation of one of those glands, particularly in childhood, I am strongly disposed to think, and I am confirmed in the opinion even by the history of the very cases which I have adduced. That those glands are only occasionally found in this situation I admit, and hence probably the rare occurrence of this particular form of disease; but that they do exist more frequently than is generally imagined I am equally certain; and I also believe that those affections of the throat termed scrofulous, when engaging the back of the pharynx, and presenting deep ulcerations, are often no more than chronic suppuration and ulceration of them.

To sum up then I would say, that I consider this affection of the throat in children, when *acute* in its progress, as, often, an inflammation of a lymphatic gland situated at the back of the pharynx; an inflammation extremely rapid in its progress to suppuration from its particular position; that I would watch for it during the period of difficult dentition, and in the several cutaneous affections or diseases of the gastro-intestinal mucous membrane to which children are liable; and that I would consider as strongly pathognomic of its presence the following symptoms:—

Fever, more or less sthenic in its character, according to the peculiarity of constitution of the child, is always present, and, I think, precedes the development of the local symptoms.

These local symptoms are premonitory and essential.

The *premonitory*, indicative of *local* uneasiness, but yet common to all affections of the throat; complained of, or otherwise, according to the age of the child, and on examination not accompanied with proportionate visible lesion. The *essential*, often very suddenly supervening, and indicated by derangement of the cerebral, circulating, and respiratory systems, alternating with the comparatively healthy condition of those systems, according to the alteration in the position of the individual. Fixed and retracted state of the head, with rigidity of the muscles at the back of the neck, and more or less locked state of the jaws. Painful deglutition, impossibility of swallowing solids, and fluids convulsively darted forward through the mouth and nose.—Repeated acts of deglutition without the presence of any fluid in the mouth, and on examination of the fauces, a firm, projecting tumor felt beyond the base of the tongue, and if seen, presenting a smooth, rounded, highly vascular appearance behind the soft palate, usually occupying the median line, but occasionally inclining to either side. These *essential* symptoms accompanied with the ordinary characteristics of suppurative fever.

The presence of those symptoms appears to me so conclusive of a collection of matter at the back of the pharynx, that I would not for a moment hesitate to decide on its nature, and proceed to open it. In such cases I think the interference of the surgeon absolutely necessary, not alone from the fact of certain fatal results from mechanical pressure on, and interference with, vital organs, but also from the situation of the abscess being particularly favourable to extensive diffusion. In opening it, great caution must be observed, and a careful assistant be at hand to steady the head, and throw it forward the moment the abscess is punctured.

I am disposed to recommend an instrument much on the construction I have alluded to. I think it a manageable instrument, and fully within control, and I think the triangular wound made with it less likely to heal by adhesion, than one made with a lancet or bistoury. Another advantage also arises from the valvular shape of the opening, whereby a too copious or sudden discharge of matter is prevented, and a greater or less extent of subsequent ulceration secured, by which the opening becomes gradually proportioned to the contracting walls of the cavity, and hence is placed in a position more favourable to permanent healing.

The necessity for caution will be proportioned to the situation of the abscess, being more called for where it deviates from the median line, or exists below the level of the tongue. I have seen one instance, referrible, I think, to this class of disease, in which a spontaneous opening took place, and here the situation of the abscess was very high up, and the discharge was principally through the nose.

It occurred in a boy not more than *four weeks old*, who had a well-marked attack of erysipelas of the face and scalp, ushered in by a severe fit of convulsions. He was under the care of my friend Dr. Fitzpatrick, with whom I was in attendance. Independent of the age of the child, the case was most unpromising, from his extreme delicacy of constitution. Every, the most unfavorable symptoms, were present. In fact, we thought the child almost in *articulo mortis*,—when a *sudden and profuse* purulent discharge took place from the nostrils. The features of the case rapidly altered, and the child got well. At that time I was unacquainted with the form of disease in question; but I think it more than probable, that it was one of those critical depôts behind the pharynx; at all events the case is worth recording, from the occurrence of erysipelas in the situation mentioned, at that very early period of life, and from its successful issue.

It is unnecessary to dwell on the diagnosis of this affection of the throat, or allude to those diseases with which it may be confounded, their respective descriptions being so extremely accurate, as merely to require reference to the authors who treat on them. Unquestionably, in the advanced stage of it, where the tumor is considerable, and the symptoms of cerebral compression intense, particularly where much debility and emaciation are present, (not unlikely concomitants,) it is more than probable that it may be overlooked, and the fatal result attributed to other causes,—and in the earlier stage many symptoms exist not unlike those attendant on spinal disease in this portion of the cervical region. Attention, however, to the history of the case, to the presence of fever, and to those features which have been noted *as essential*, will at once remove any difficulty.

*Chronic* abscesses, single or otherwise, are also occasionally found in this situation during the earlier periods of life. They are often actually formed of some size before detected, and this, probably, from the little inconvenience they occasion. They are connected more or less with that state of constitution termed scrofulous, and I am satisfied they will be found of the same nature with similar degenerations (if I may use the expression) of the cervical glands, so common in those constitutions. The symptoms attendent upon them are in a much milder degree of the same character with the acute, and perhaps, the most prominent are, the remarkable effect produced on the respiration by change to the recumbent posture. There is absence of fever, and throughout the day the child is free from any obvious illness,—able to play, and join in the amusements of other children; I have known them not to complain of any uneasiness in the throat, and attention to be directed to it, from the raucous breathing during sleep. In fact, the symptoms much resemble those of common scrofulous induration of the tonsil. They are hence cases of comparatively minor importance; there is time to investigate them. Indeed, with them may be complicated chronic enlargement of the tonsils. I have met with them after scarlatina, after variola, and after measles. In fact, they are some of the sequelæ of those cutaneous diseases, and like them may be accompanied with suppuration of the internal or external ear, and so come under the description of similar cases already alluded to, as described by Petit. They possibly may require surgical interference, and always are benefited by those local and general remedies suited to their peculiar nature. At the same time, it is perfectly intelligible that they may undergo a spontaneous cure, and altogether escape observation.

The remarks which I have as yet made are principally in reference to this affection, as an occurrence in childhood. From them the following conclusions are, I think, justly deducible.

First,—That a new cause of obstruction in the throats of children exists independent of those noted by authors who treat on their respective diseases.

Secondly,—That the presence of the obstruction is indicated by symptoms peculiarly characteristic, although extremely equivocal in their nature, if not accurately investigated. And

Thirdly,—That its removal is effected by an operation, simple in performance, and, as far as can at present be ascertained, certain in its results.

With respect to this affection in *adults* I have already stated that I have not had an opportunity of witnessing an instance. I cannot, however, imagine otherwise, than that the attendant symptoms, in the incipient and advanced stages of the inflammation, must very much resemble those as detailed in the child, with the great additional advantage of the history from the patient, and yet, how extremely anomalous and deceptive must they not be, when we reflect on the cases which I have brought forward and reported. In all, the *premonitory* symptoms did not attract attention, and even in those which were recognized, and terminated favourably, the *essential* were too characteristic, from the extreme developement and mechanical operation, to escape notice. That they occur, however, cannot be questioned, and that they are, with certain restrictions, attributable to similar causes, is more than probable.

The case I have alluded to in the "Dictionnaire de Chirurgie, &c.," is conclusive as to the occasional supervention of abscess behind the pharynx during or after fever, in the *adult*, just as the first case I have detailed, is, in the *child*.

"Il est assez rare que l'on soit obligè de porter l'instrument tranchant dans le pharynx, soit afin de donner issue à des collections purulent formèes à son voisinage, ou dans son epaisseur, soit pour dégager et extraire des corps ètrangéres arretées dans sa cavité. J'ai vu, cependant, il y a peu d'années, un malade qui, a la suite d'une gastro encéphalite grave, avait eu un *abscès critique volumineux* à la partie posterieure du pharynx. La bouche ètant *largement ouverte*, et *la langue abaissée avec le doigt*, ou *le manche d'une cuiller*, la tumeur se présentaite rouge, tendue, luisante et lisse à sa surface, faisant dans le pharynx une saille considerable, qui obstruait cette cavité, rendait la respiration difficile, s'opposait au libre passage des substances alimentaires, et alterate beaucoup l'articulation des sons. La tumeur s'étant développée dans la paroi cervicale du pharynx, directement en arrière de l'isthme guttural, l'incision qui en fut pratiquée donna issue à une grande quantité de sus. qus le malade rejetta au moyen d'efforts d'expulsion peu considerables, et la guèrsion eut lieu en peu de jours."

In this case and that given by Sir Astley Cooper, it is remarkable with what facility the mouth was opened, and the tongue depressed, as it forms a strong contrast with the almost utter impossibility of accomplishing the one, and the excessive pain induced on attempting the other, in those cases I have met with. It may perhaps be attributable to the greater powers of accommodation of the structures engaged to their new position, at the former period of life than in the latter; but I am inclined to refer it more to the very rapid progress of the acute form of the disease in children, and the accompanying fever, a circumstance not noted in the other cases. They are, in fact, more of a chronic character. Indeed no account is given of the constitutional symptoms, or whether any existed; and it is almost certain that such would not have escaped the accurate research of those who witnessed and reported them, did they present any peculiarity beyond that arising from their local effects. Hence an additional circumstance in favour of their *chronic* nature.

It would certainly be a most interesting fact to be enabled to adduce an instance of the occurrence of such an affection, *acute* in its progress in the adult. Possibly some of the suddenly fatal cases in tonsilitis are confounded with them.

Allan Burns, in his "Surgical Anatomy of the Head and Neck," thus expresses himself when on the subject of suppuration in the tonsil.*

"When the collection of matter is large before the abscess bursts, the patient is in a more dangerous situation than is generally imagined. His breathing is obstructed and gasping; he feels much anxiety in the chest; his face is dark and bloated; his eyes are painted with vessels containing purple coloured blood, they are prominent, and seem ready to start from their sockets. We cannot be deceived in regard to the origin of those symptoms, which decidedly show that the lungs are imperfectly supplied with impure air. Whenever the abscess bursts, the mouth and fauces are filled by a gush of matter, every obstruction to the free entrance of air is suddenly removed, the patient fetches an involuntary and deep inspiration, air and matter rush together into the trachea, and death from suffocation is almost the inevitable consequence.

"This, to some, may have the appearance of a fanciful description, or, at all events, an overcharged picture; but its fidelity will be admitted, when I inform them, that in this very way a strong, active young man lately lost his life. He had been complaining *for a few days of a sore throat*, for which he had consulted his surgeon, who had employed the usual remedies. The inflammation terminated in suppuration: the abscess enlarged until the tumor occupied the entire fauces; yet *ten minutes before his death*, he was walking about the house, restless indeed, anxious, and gasping for breath. The bursting of the abscess, and death, followed each other so rapidly, that no measures could be taken to prevent the latter event.

"The cause of death was not conjectured in this instance: the body was examined, *and the trachea found deluged with purulent matter.*"

It is much to be regretted that the condition of the larynx is not particularly noted, as the history of the case is by no means conclusive of the actual nature of the affection of the throat; it merely states, "sore throat"—a very equivocal expression.

Is it not a fact, that in the most severe cases of acute cynanche tonsillaris, the inflammation occupies a greater or less portion of the soft palate and its pillars? That the tongue can be hardly protruded, and that the jaws are separated with difficulty; and in this condition is not the base of the tongue so circumstanced as rather to favour the protection of the glottis; an office in which it is not unlikely assisted by the effusion of serum, to a greater or less amount, on the anterior aspect of the base of the epiglottis? Here is it likely that an abscess of the tonsil would burst into the trachea? or rather that the glottis would admit the matter? I think not. I think in such severe

* Surgical Anatomy of the Head and Neck, by Allan Burns. Glasgow Edition, 1824.

cases death is much more attributable to the surrounding serous effusion producing œdema of the glottis, and its consequences; or to the extension of a bad character of inflammation producing a similar effect on the glottis, from sub-mucous purulent infiltration.

It may be said that among the symptoms I have enumerated as pathognomic of abscess behind the pharynx, the peculiar state of the jaws now noted, existed, and that it equally favours the same position of the tongue, and the same condition of the glottis; but here it must be borne in recollection that the situation of the abscess (when of a *phlegmonous* character, perfectly *circumscribed*) would above all others oppose this effect of the epiglottis. It acts from *behind forwards*, and so far forms an obstacle to otherwise perhaps fatal results, an obstacle assisted within certain limits by the posture selected by the patient. But on the other hand, how is this effect circumstanced as far as regards the glottis, should this abscess give way, or should the surgeon be incautious in opening it? Surely the passage of the matter into the trachea is almost inevitable. May such results not have occurred without detection? may it not have been the case here? The only provision against such a termination is the extreme laxity of connexion between the spine and the posterior part of the pharynx favouring its descent, and the more dense nature of the aponeurotic expansion already alluded to *in the median line*, being unfavorable to its pointing in that situation.

When we recollect, however, the laws adopted by nature to direct the progress of abscesses situated near *mucous* membranes, and that those laws are more strictly adhered to, the more *acute* the nature of the abscess, and the more distant from a *cutaneous* surface, we can reconcile to ourselves the opinion that such provisions would not prevent the *direct* bursting of the abscess, notwithstanding their presence. The same remarks are not applicable to *chronic* abscess; and hence the extended route they take may be accounted for, as instanced in two cases reported in the Transactions of the Association of the College of Physicians, on abscesses between the *œsophagus and spine*, which at their commencement were most probably situated behind the upper part of the pharynx, and there recognizable by sight or touch.

In the adult, then, as in the child, the acute abscess behind the pharynx imperatively calls for the early and prompt interference of the surgeon, who must necessarily observe the same caution already alluded to in reference to its treatment. The selection of the trochar is perhaps still more advisable, from the great probability of a considerable accumulation of matter. The constitutional treatment here, as in that of the child, includes, of course, those restorative means generally adopted under similar circumstances.

Chronic abscess in this situation is, in the adult, I would say, always symptomatic of some constitutional derangement resulting from a specific taint.

It may be scrofulous, and decidedly it may be connected with those anomalous affections occurring in the progress of cases of a pseudo-syphilitic character. As, in such effections elsewhere, our principal aid must be derived from constitutional treatment, which it is unnecessary here to particularize. The local treatment may vary in each individual case as to time of interference, but in all I believe the slow evacuation of the contents of the abscess is prudent. The complication of an abscess in this region of the spine with disease of any of the cervical vertebræ, will of course materially modify its character and progress, but not having met with such I merely allude to their possibility of occurrence.

The diagnosis of this affection in the adult is to a certain extent unattended with much of difficulty, and principally, perhaps, as in the child, escapes detection, from the little local distress induced by its presence. Some caution, however, is called for notwithstanding, as in the situation in which it occurs other affections of a chronic character are met with, with which it may be confounded. Allan Burns, for example, mentions a case where a *polypus* was mistaken for an abscess of the tonsil. Is it not equally probable, that a similar mistake may be made in the case of chronic abscess behind the pharynx; or, might not a chronic tumor, malignant or otherwise, in the same situation, lead to a similar mistake? These considerations, however, are not exactly relevant, or if so, are unnecessary, as they imply more than unusual carelessness on the part of the practitioner.

Enough, then, has been advanced, confirmatory of the presence of this form of disease in children, and in adults, and explanatory of those most prominent symptoms attendant upon its progress and full developement. It most certainly is to be met with at *both epochs* of childhood, as an *acute* and *chronic* affection, and more than probably is similarly so in adult life. In both it requires on the part of the surgeon promptness and extreme accuracy of diagnosis, and in both the most circumspect caution and decision as to treatment. Attributable to causes already specified as generally applicable, the chronic form in the adult may be complicated with constitutional lesions somewhat peculiar, arising from diseases to which he is more liable.—*Lon. Med. Gaz.*

---

*Observations on the Premonitory Symptoms of Insanity, with Cases.* By John Grantham, Esq., Surgeon.—The object of this paper is to detail the premonitory symptoms of insanity, and especially such as present themselves in the generality of mental diseases. The moral effects which demand attention may be classed under ten heads (and let me premise by asserting that all these moral effects may arise *pro tempore* without any fear of insanity; nevertheless I feel warranted in regarding them as worthy of attention in combination with the general history of the case:)

1. Undue suspicion,
2. Discontent,
3. Remorse,
4. Disaffection,
5. Revenge,
6. Indolence,
7. Excitement,
8. Unnatural activity in the pursuit of different objects,
9. Fearful apprehension,
10. Forgetfulness.

Not that I am intending to enter into any controversy on the merit or the demerit of phrenology, yet in this inquiry I feel it due to my own sense of justice to advocate the right use of the system in the investigation of moral causes. Phrenology teaches, first, that the brain is the organ of the mind, and is concerned in every mental operation, whether of emotion or intellect; secondly, that the brain does not act as a unit, but consists of a plurality of organs, each serving for the manifestation of an individual faculty of the mind; thirdly, that the energy of function, or power of manifestation, is proportioned *cæteris paribus* to the size of the organ; or, in other words, that a large organ *will, all other conditions being equal*, enjoy a power of action proportioned to its size, and, consequently, manifest the corresponding faculty with greater energy than if it were small. Cuvier says, "l'anatomie comparée en offre one autre confirmation dans la proportion constante du volume de ces lobes avec le degré d'intelligence des animaux;" thus admitting the influence of size of the cerebral organs upon the power of manifesting the mental faculties as distinctly as Dr. Gall himself could assert it. But, to prevent any misrepresentation, let it be observed that there is scarcely a phrenologist who does not utterly scout the notion of organic size being the only condition of functional energy. To demonstrate the evidence of organic size being *cæteris paribus* a measure of functional power, let any one look into the field of nature, and there examine the testimony of exery anatomist and physiologist who treats of the relation between structure and function. The brain, in its functions, must be relatively studied in reference to the amount of muscular power in the body. Dr. Marshall Hall states, "That the cerebrum is, in its acts of volition, an exhauster of muscular irritability; that in muscles separated from their nervous connexion with the brain we have augmented irritability." It is admitted that insanity begins in the slightest departures from healthy feeling, and may be traced through every variety of shade to forms of severity, in which it is so evidently associated with an infirm, ill-judging, ill-reasoning, and perverted mind.

After examining the moral effects, the next step will be to ascertain the primary cause, and which will be found in the deranged function or diseased action of one or more of the various organs of the body. To descend to minutiæ here would only be a tedious repetition of those pathological symptoms well known to the reader; yet it may be well just briefly to observe that the skin must be noticed with regard to its functions as an absorbent, exhalent, and regulator of the animal heat; the muscles, as to their action in reference to volition; the head, in reference to action and power; the blood, as to its condition and composition; the assimilative organs, in reference to diet and muscular power of the stomach and large intestines; and the glandular system, in reference to the supply of the nerves of organic life.

As far as I have prosecuted the diagnosis of the premonitory symptoms of insanity I have invariably found the exciting cause to exist in the spino-excito-motory system, and by sympathy to the brain. The primary cause or causes come under the appellation of depression, stimulation, and irritation. The fever that generally attends the early stages of insanity is of the congestive character. Congestion of the venous system is a state produced by the operation of common depressants, and marked by a diminution of the animal heat on the surface of the body, a diminution of the heart's action, and by a disturbance in the function of this or that organ which is the seat of congestion. The pathological inquiry must also be continued, first, in reference to predisposition; secondly, to disorder or deranged function; and, thirdly, to diseased action. The medical treatment called for in each case in which the mental disorder depends chiefly or entirely on some disordered condition of the heart, the liver, the stomach, the intestines, the uterus, &c., must, of course, vary in each particular case, and be conducted on general principles. In exemplification of the treatment it is my intention to publish those cases which may, in addition to the following, come under my observation.

*Case* 1.—S——l M——n consulted me March 31st, 1840; he was about twenty-six years old, of a middle stature, in temperament sanguineous. He complained of lowness of spirits, and inability to perform any of his usual duties; he lost all confidence in himself, and thought every one regarded him with distrust; his religious views became unsettled, and his confidence in the divine promises disturbed by the fears of his final perdition, which he thought must be his inevitable doom. He was a man of the strictest integrity, and esteemed as such by his employer, naturally of a lively and amiable temper, &c., &c. He left his business, which was that of a clerk in a notary's office, in London, in the month of November, 1839; he felt himself unwell, and consulted his medical adviser, who, considering he had simple fever, made no restriction of diet or action: in a fortnight he resumed his employment, but the malady returned with increased severity: he a second time consulted his medical friend, who termed his case a nervous affection, and placed him under a tonic treatment of aromatics, camphor, &c., and wished him to leave the neighbourhood of London; before going away he was advised to have the opinion of another medical man, who approved of the plan pursued, and also stated it was necessary that he should leave town; he then went to the Island of Guernsey, and there sank into a state of deep melancholy: he there consulted a physician, who told him to take much exercise, walk about, but ordered no medicine, stating it as useless. From thence he went to Jersey, where he suffered more depression of mind. After remaining some time in the latter place without benefit, he returned home, and a third time consulted his family medical attendant, who advised him to take a voyage to New York, but ordered no medical treatment: having a great reluctance to this advice, he came into the country to be under my direction. On examination he stated his inability to rest, with occasional dimness of vision, taste depraved, hearing good, the bowels inactive, urine pale, but unable to expel the last drops; muscular action defective, the skin dry, with occasional sensations of heat and cold, not general, but partial. On inquiring as to the original cause, I learned he had been washing his feet in cold water at night just before going to bed.

*Treatment.*—I ordered him to have the feet put into a hot bath at 100 degrees, composed of six tablespoonsful of mustard in two gallons of boiling water, for fifteen minutes, at bed time; to use the flesh-brush to the skin in the morning; to remain quiet: the diet to consist of gruel for breakfast, mutton broth or beef tea, with bread, for dinner; no tea, and a gruel or arrow-root meal at seven o'clock in the evening; to abstain from fermented liquor and solid meat; to take twice a day Pil. Hydr. gr. v. and a Draught composed of Carbon. Sodæ, ʒj.; Spt. Ammoniæ Co. ʒj.; Pulv. Rhei, gr. vj.; Misturæ Camphoratæ, q. s.

*April 2d.*—He stated he had slept the whole night, such a circumstance as had not occurred during his illness. I requested him to continue the measures as stated—to take walking exercise, so as not to cause fatigue. I scarcely need observe that the partial paralysis of the muscles of the perineum were owing to defect in the internal pudendic nerve, which is supplied from the fasciculi of the fourth and fifth lumbar and three upper sacral nerves; this will lead to the exciting cause that the spino-excito-motory system was deficient in its supply of the power of organic life to the large intestines, kidneys, and, probably to the mesenteric glands, and through the medium of the muscular deficiency, exhausting the power of the cerebrum.

*4th.*—The animal heat more equally diffused; continues to sleep well; appetite improving, and feels less gloom of mind. Continue the medicine.

*6th.*—Improving: the evacuations becoming more healthy, and the skin inclined to perspiration towards the evening. Continue the medicine.

*8th.*—Still improving, especially as regards the mind, but feels great general debility; continue the pills until gentle ptyalism is produced; increase the diet: an egg with the breakfast. This system was continued so as not to produce prostration of the animal powers, increasing the diet as the mental faculties became natural, enjoining moderate exercise and mental quietude, avoiding excitement, until

*May 14th.*—The weakness now being confined to the back over the region of the fourth and fifth lumbar, and three upper sacral vertebræ, and legs, I requested him to use a cold shower-bath every morning, and resume his duties, which he has done, and is improving in strength up to the date of this paper, not having had a return of his melancholy feelings. The above case is an example of the good effect of the mercurial action combined with the alkali, (carbon. soda:) the former being an excitant and antiphlogistic, while the latter acts as an antiseptic.

*July 7th.*—I have heard he is deriving immense advantage from the cold shower-bath as a tonic.

*Case* II.—Wm. K——p, a painter and publican, applied to me in the year 1828: a stout, middle sized, dark complexioned man, he complained of pain and weight over the head, imperfect vision, loss of appetite, great debility, pulsatory sensation across the occiput, trembling of the hand, evidently from intemperance; his feelings were low and desponding; he wept on the slightest occasion; he wished for solitude, and yet feared to be alone, either suffering the most dreadful nervous irritability or sunk in the deepest despondency. The treatment consisted in enjoining an abstinence from fermented liquor, loss of about twelve ounces of blood taken in a full stream from the arm, and repeated mercurial aperients, which had the effect of restoring him to health of body and mind. From the above time to Dec. 28th, 1839, I lost sight of him, when I was sent for in haste, he having attempted suicide, and had divided a portion of the right parotid gland, and some arterial branches of a moderate size, so as to bleed rather profusely: since I saw him he had very much increased in flesh, from indolence of habit, as he had been out of work for some time, in consequence of an irritable disposition rendering him obnoxious to his employer; he had again drank freely of both beer and spirits. The membrana conjunctiva was inflamed; the pulse one hundred and twenty, and tremulous; pain in the hepatic region; distension of the stomach and large intestines; uneasy sensation over the whole of the dorsal vertebræ; paralysis of the right side of the tongue; neuralgic pains down the right side of the neck; urine scanty, high coloured, depositing after standing a brownish sediment. After dressing the wound I ordered the patient to be kept on low diet, to remain in bed, and to take the following draught every four hours:—

℞. Magnesiæ Sulphatis, ℥iij.; Antimonii Tartarizati, gr. i.; Inf. Sennæ c. q. s. M. Haust. ℥iss.

*Dec.* 29*th.*—Had a restless night; very incoherent in his talk; pulse full. I took sixteen ounces of blood from the arm; ordered the aperient draught to be continued, with Pil. Hydr. gr. v. added to each dose.

30*th.*—Slept about two hours; bowels acting freely, the evacuations dark and very offensive; tongue white, and the edges uneven. Omit the Ant. Tart., and take a teacupful of beef tea in the day.

31*st.*—The pulse ninety; more natural; the mind is becoming tranquillized; the vision still imperfect; complains of a noise in the ears, with tenderness in the right side. I now determined on continuing the mercury until gentle ptyalism ensued, with a spare diet, perfect quietude of mind and body, forbidding all conversation. The state of mental stupefaction in which he was when he committed the act was after a few days succeeded by feelings of the bitterest remorse and contrition; nothing could pacify his self-accusations for having attempted so horrible a deed, until I explained to him that his crime was itself an almost involuntary act, depending upon his deranged health, and that it was his previous ill conduct and intemperance which had induced the attempt: he said "he had not been well the last six or seven months past, frequently wandering he knew not where, until hunger or fatigue roused him to reflection; and at other times shunning the society of friends, and stupifying himself in the corner of some public-house-tap room, imagining that every one distrusted him, and regarded him as unworthy of their friendship." My reasoning with him in endeavouring to convince him that health of mind was dependent on health of body, seemed to inspire him with hope, and allowed of my furthering the medical treatment, which was continued by attention to the sanguineous system and prima viæ, by removing the inflammatory condition of the membranes of the brain, with the mercurial and alterative treatment.

*Feb.* 14*th*, 1840.—I took my leave of him, after assuring me how gratefully he felt for his restoration to health of mind and body.

*Ibid.*

---

*Researches into the Pathology, Physical Signs, and Treatment of Pericarditis; illustrated by Cases.* By THOMAS MOORE, Secretary to the Dublin Medico-Chirurgical Society for 1840–41.—In most of the cases hitherto detailed we have exemplified, in greater or less perfection, the symptoms usually described in books as attendant upon, and characteristic of, the onset and progress of this disease. Permit me now, gentlemen, to occupy your time a few minutes longer, in mentioning a case wherein an attempt was made to counterfeit this complaint, not so much for the instruction it will impart, as to exhibit the schemes soldiers will sometimes resort to in order to avoid the punishment due to their faults.

A private in the 22d regiment, having obtained furlough for three months, returned from the country, and remained in this city till the 15th July, the day of its expiration; by applying at the Town Major's Office for an extension of a few days, he was granted five, to enable him to proceed to Chatham, where the depôt of his regiment was then stationed. Instead of availing himself properly of this indulgence, he remained in this place, disregarding future consequences, revelling in drunkenness and debauchery. Thus misspending his time, as soon as the period allowed had elapsed, he again made application, but received a severe reprimand, and was put under arrest.

That night, whilst in the guard-house, he alarmed his comrades by his frequent groanings, and occasional loud shrieks, complaining of his being on the point of suffocation, and in the greatest agony from pains in the chest; a report of his state was then made by the serjeant to the officer of the guard, who immediately ordered him off at an early hour in the morning to the Royal Infirmary. The next day he was seen by two or three persons connected with the hospital, and the following report taken down:—Extremely rapid and seemingly distressed breathing, amounting to fifty-five in the minute; distorted features, and considerable anxiety about the countenance; insatiable thirst; the history containing all the symptoms indicative of an attack of fever; his skin was moist and perspiring; the tongue clean and moist; all his pains were referred to the left side; the slightest attempt at drawing in a full breath produced inconceivable distress, and a few hours before he had had violent beating and palpitation of the heart; when the hand was placed over the cardiac region, and a slight degree of pressure made, he roared aloud, so that all the other patients imagined the person was guilty of great cruelty; the mere application of the stethoscope alone caused the same degree of suffering; the pains he described as being now here, now there, shooting up and down the side; from the rapidity of the breathing it was impossible to ascertain the state of the internal organs; his pulse, notwithstanding all his aches, examined several times, varied from seventy-six to eighty, but never exceeded the latter; was full and compressible. He was ordered to be bled in the arm to twelve ounces.

Being deputed to bleed him, conjecturing, from the character of the pulse and the circumstances connected with the history, that he was acting a knavish part, I was determined to treat him, as all melingerers should; accordingly, having got ready the lancet, and tied up the arm, there was an evident repugnance on his part to hold it out, uttering many objections and excuses; he several times refused to allow the vein to be punctured; at length, after much ado, having got the arm into a proper position, I made a large opening, the blood flowing out in a full stream to his great discomfiture: at the time that this was in performance his respirations also were accurately watched and counted; twelve ounces were taken, no amendment in his symptoms; sixteen ounces, not the slightest; finds it quite impossible to draw in a full breath; the respirations vary from fifty to fifty-five; but is grumbling exceedingly at this mode of treatment, saying, "it will do him no good;" in a few minutes more he had lost twenty ounces, but without relief; his breathing, however, is not so accelerated, having fallen to forty; he is also commencing to perspire about the face: as soon as twenty-two ounces were abstracted, he complained of weakness, sickness of the stomach, and an inclination to vomit, but still persisted in saying, "he was as bad as ever;" at last, after obtaining twenty-four ounces, and when on the point of fainting, he exclaimed, "Stop, sir, stop, I'm better;" the respirations now did not exceed twenty-four, and pressure to any amount might be made over the heart. The next day he had the same catalogue of complaints, being quite certain that bleeding would not be ordered, and quite determined to refuse should it be prescribed; a bolus of the most drastic purgatives was made up, which afforded but little rest during the remainder of that day and the entire night; the next morning he was informed that any further complaints on his part would not serve him in the least, as they would not be attended to, whereupon he demanded to be sent to his regiment, "where he would be treated far better than in a place in which he was one day almost bled to death, and the next had his guts purged out."

Although this treatment may appear very harsh to those unacquainted with such characters, yet it was the only one likely to subdue and overthrow his preconcerted plan. Had a more lenient plan been pursued, instead of troubling the hospital for three or four days, he would have converted it into as many months, and, probably, would have deprived his regiment *in toto* of the services of an able-bodied and efficient soldier.

I shall now close this treatise by forming a comparison between the several cases contained in the foregoing pages :—

1. In the first of these, the disease we speak of was secondary to, and consequent upon, another, being of the most common form usually described, viz., rheumatic pericarditis; whilst in the others, we have every reason to think it was primary or purely idiopathic, originating in some external exciting cause.

2. The first, though greatly debilitated from a description of arthritis, was under our immediate observation, and had the disease treated almost at the very onset; whilst the others, possessed of constitutions unravaged by previous diseases, were treated for their complaints when at a very advanced period.

3. The one (Kennedy) was combined with pleuritic affection; whilst in the others, (the last case excepted,) though the action was weak and irregular, yet the sounds retained their due proportion to each other, rendering life still more precarious, and our prognosis unfavourable; the others, uncomplicated, but without better expectations.

4. The pulse, in the first case, was full and regular; in the others, feeble, irregular, varying in strength and intermittent, there being in general a strict concurrence between the character of pulse at wrist and the action of heart, heard in præcordial region.

5. The jugular veins in all four were distended from the obstruction afforded to the return of the blood, confined to or more remarkable on the left side; possessed of an undulatory motion in the two men, whilst in the little girl this did not appear to exist; whereas in the case lately seen, nothing could be more obvious, so much so, that the pulsatile motions appeared derivable from the expansion of the pulmonary texture by the entrance of air.

6. There was dulness with the other phenomena of pleuritic effusion in the left pleural cavity of one; whilst, in the others, an extraordinary clear, almost tympanitic, sound was observable: the respiration in the former being intensely bronchial; but in the latter, of that nature which may be defined non-terminal.

7. In the coppersmith's case, the supra-clavicular tumour extended much higher in the neck, was more prominent, and disappeared after the absorption of the effusion; in the last case it was equally prominent, the displaced or protruded lung mounting nearly to the same height, but remaining unchanged in its position; whilst, in the other two, the phenomenon was not quite so distinct, but sufficiently so to attract our attention, and continuing conspicuous till the death of the patients. Lastly, the first sound of the heart, in the coppersmith, appeared to have been affected like that in typhus fever described in Dr. Stokes' paper, with the morbid poison then prevalent in the system, manifesting, before and after the attack of pericarditis, that the muscular tissue must have participated in the general debility of the system.

The conclusions, which may be inferred from the preceding cases and their attendant phenomena, may be enumerated as follows :—

1. That the appearance of a tumour, extending from the left clavicle upwards into the neck; a tumefaction or puffy state of the integuments above this clavicle, contrasted with the right side, and attended with a clear sound on percussion; the existence of respiratory murmur, &c.; the elastic, sometimes downy, sometimes crepitating feel on pressure, with an increase on coughing, should induce us to suspect displacement or protrusion of the lung upwards.

2. That when these appearances and physical signs persist for a length of time, whether the symptoms have or have not subsided, the probability is, that adhesions have taken place between the pleura and surrounding parts; but the greater likelihood is, that, with the removal of the disease, the return of the displaced lung will take place.

3. That displacement of the lung upwards, forming a supra-clavicular tumour, was, and may be, a most valuable assistant in enabling us to determine the existence of pericarditis with effusion, when combined with pleuritic effusion on the left side.

4. That a full and puffy state of the lower portion of the left side, contrasted with the right, and attended with the phenomena recorded in the above description, may likewise materially assist us in detecting pericarditis, uncombined with pleuritic effusion.

5. That in uncomplicated pericardial effusion, there exists another curious, interesting, and valuable physical sign—explain it as you please—namely, the clear sound on percussion, in some cases confined to the posterior part of the left lung; but when the fluid is in enormous quantity, observable over the right; there still existing an evident difference between the sounds afforded by both sides.

6. That permanent, or occasional turgescence of the jugular veins, with or without an undulatory motion, is also an attendant phenomenon on this form of disease, and seems to depend on pressure of the lung against some of the large veins; the pulsations or retrograde motions of the blood appearing to be produced by and during the expansile impulses of the lung, inflated with air; retarding the onward flow of the current, and communicating to it a shock, as manifested by the undulation of the blood.

7. That all these, in connection with others, will assist in forming a group of signs, which will render the presence of fluid in the pericardium still more easily diagnosed, but little importance being attached to them when taken separately.

8. That in some cases of pericarditis, and in others unconnected with this disease, there is often present a peculiar "clicking sound," or the succession of a number of "clicks," the cause of which is at present inexplicable.

Lastly. That in many cases of pericarditis, we may often be enabled to infer its supervention, prior to any of the ordinary train of symptoms or physical signs having been developed, by observing the state of the pulse; and that this remark is more applicable to patients affected by rheumatic arthritis, whenever a regular intermission in the pulse occurs.—*London Lancet.*

---

*Successful treatment of Dropsy of the Synovial Membranes by Tartar Emetic.* By M. Gimelle.—M. Gimelle has found the administration of tartar emetic in large doses very efficacious in curing dropsy of the synovial membranes; causing complete absorption of the fluid, with abatement of all inflammatory symptoms, if any exist. Twenty-seven cases of dropsy of the joints have been treated successfully by him. He, without any previous treatment, commenced by giving four grains of tartar emetic in the twenty-four hours, and increased the dose by two grains every day, till from eighteen to twenty grains were taken daily. As soon as toleration of the medicine was established, the fluid began to be absorbed, and the cure was in general complete in from eight to sixteen days.

In only five of these cases was vomiting excited by the medicine, in two cases for three days. In eight cases it produced alvine evacuations; but its most general and constant effects were diminution of the strength and quickness of the pulse, weakness of the voice, abundant nocturnal perspirations, and the appearance of a dark circle around the eyes. In almost every case the appetite remained unimpaired. M. Gimelle regards this plan of treatment as the most successful ever yet proposed for the treatment of dropsy of the synovial membranes.—*Ed. Med. and Surg. Jour.—from Bulletin de l' Academie Royale de Medicine,* 4th July 1840.

---

*Two Cases of the Excision of the entire Clavicle: one operation performed in* 1835 *by Dr. Cajetan Mazzoni, of Pisa, the other by Professor Charles Biagini, of Pistoia.* Communicated by Dr. Aronsohn, of Strasburg.—When we consider the situation of the clavicle and the important offices it fulfils, it is natural to suppose that its loss must be followed by severe inconveniences, and that the form of the chest and shoulder with the motions of the arm would be necessarily injured. Experience, however, in this as in other cases shows the futility of *à priori* reasoning in medicine.

Case I. The first case was one of necrosis of the clavicle in a boy four years of age, of a decidedly scrofulous habit. About the time when spontaneous separation of the necrosed portions of the bone was commencing, Dr. Mazzoni considered that the time had arrived to assist nature by extraction. Believing that the attachments of the clavicular muscles were destroyed, not only because necrosis was made evident by the probe, which proved that the periosteum was gone, but also because the bone was moveable, Dr. Cajetan thought it only necessary to divide the ligaments situated at the articular extremities of the bone. A director was introduced into a fistulous opening which existed near the sternoclavicular articulation, and a bistoury being passed along it the integuments were divided: the clavicle was seized with a pair of forceps, and the costo-clavicular ligament was divided with a straight blunt-pointed bistoury. Afterwards the other ligaments uniting the clavicle to the sternum were divided. The acromial extremity of the clavicle was apparent through an ulcer of the integuments, and therefore this bone was easily detached from the scapula. The entire extraction was completed with little loss of blood, and a portion of integument between the two wounds remained entire. The cure was completed within a month.

Three years after the operation the patient was in perfect health. The shoulder deprived of the clavicle was slightly depressed and approaching the sternum, but without affecting the symmetry of the parts. The motions of the arm were unlimited in any direction, the patient readily climbing trees.

Case II. A boy fifteen years of age was admitted into the hospital of Pistoia in August 1838 under M. Biagini. The clavicle was affected with scrofulous disease, and was removed in a similar manner to the former case. Slight hæmorrhage followed; but was stopped with charpie and moderate pressure. Caustic was occasionally applied to the wound and it had cicatrized within thirty days. This case is peculiarly interesting, as a firm fibro-cartilaginous substance now takes the place of the lost clavicle, so that the loss is not felt, every motion being perfect.—*Brit. and For. Med. Rev.*, *from Gazette Medicale. Juillet* 18, 1840.

---

*Varieties in the form and position of the Liver.*—*Varieties in form* occasionally occur, but they are more rare in the liver than in almost any other organ of the body. I have seen the left lobe so small, as to appear but a mere appendage to the right, being connected to it only by a thin and narrow isthmus. Cruveilhier records an instance in which the left lobe was attached to the right merely by a vascular pedicle about half an inch in length; the extremity of the lobe being adherent to the upper part of the spleen. Deep and narrow grooves are occasionally seen upon the convex surface of the right lobe running in an antero-posterior direction; they correspond with projecting fasciculi of the diaphragm, and occur generally in women who have laced tightly. This surface is also marked frequently in females with deep channels, which are formed by the pressure of the ribs, and are also the result of tight lacing. The liver is sometimes constricted in the middle from this cause, and a dense fibrous band, produced by thickening of the fibrous capsule, extends around it like a belt. The lobes are occasionally divided by deep fissures into several additional lobes; the liver in this case presents a character which is normal amongst the lower animals. In a few instances the fossa for the gall-bladder has been found excavated so deeply, as to render the fundus of the sac apparent through an opening on the upper surface of the liver; a peculiarity which is also normal amongst some of the lower tribes of animals.

*Varieties of position* are more frequent than those of diversity of form. During uterogestation the liver is usually pressed considerably above its ordinary plane, so as to impede more or less the action of the diaphragm, and produce embarrassed respiration. In an extremely fat subject, I once saw the diaphragm raised by the liver to a level with the fourth intercostal space, measured near to the sternum. In its natural position the thin margin of the liver scarcely reaches the border of the thorax; but in women who have laced tightly during youth, nothing is more common than to find this edge forced several inches below the base of the thorax, and altered in its form. In these cases the direction of the aspects of the organ are likewise changed; the convex surface looks directly forwards, instead of upwards and forwards, and lies in contact with the abdominal parietes. The concave surface is directed backwards, in place of downwards and backwards, and the posterior border is forced upwards. In a sketch from the subject, now before me, the greater part of the convex surface of the organ is in contact with the abdominal parietes, and the free margin extends into the umbilical and lumbar regions. In another sketch, as a result of the enormous magnitude of the stomach from the same cause, the liver is raised almost perpendicularly, the extremity of the left lobe being in contact with the diaphragm, and the right lobe in the right iliac fossa. A part of the liver has been found in the sac of inguinal and umbilical hernia. Various peculiar appearances are observed in the liver of the fœtus, arising from arrest of developement. Thus, for instance, the entire organ, or a part of it, may be situated in the chest, or from absence of development of the abdominal parietes, the liver may form part of an exabdominal tumour, and be uncovered excepting by the membranes of the ovum. But the most interesting and unexplained form of altered position is that in which the whole of the viscera of the body are transposed, and the liver becomes placed on the left instead of the right side. These cases are generally perfect, and the peculiarity does not seem to interfere with the life or functions of the subject. The liver presents its natural form and size, and with the simple exception of left for right, precisely the same relations. The aorta, of course, occupies the right side, and the venæ cavæ the left, while the stomach is transferred to the right. Sir Astley Cooper has preserved the viscera of an adult who was the subject of this transposition.—*Mr. Erasmus Wilson in the "Cyclopœdia of Anatomy and Physiology.—Lon. Lan.*

